# The Burden of Cardiovascular Disease Attributable to Major Modifiable Risk Factors in Indonesia

**DOI:** 10.2188/jea.JE20150178

**Published:** 2016-10-05

**Authors:** Mohammad Akhtar Hussain, Abdullah Al Mamun, Sanne AE Peters, Mark Woodward, Rachel R. Huxley

**Affiliations:** 1 Division of Epidemiology and Biostatistics, School of Public Health, The University of Queensland, Brisbane, Australia; 2 The George Institute for Global Health, Nuffield Department of Population Heath, University of Oxford, Oxford, United Kingdom; 3 The George Institute for Global Health, The University of Sydney, Sydney, Australia; 4 School of Public Health, Curtin University, Perth, Australia

**Keywords:** population attributable risk, risk factors, attributable risk, non-communicable diseases, cardiovascular diseases

## Abstract

**Background:**

In Indonesia, coronary heart disease (CHD) and stroke are estimated to cause more than 470 000 deaths annually. In order to inform primary prevention policies, we estimated the sex- and age-specific burden of CHD and stroke attributable to five major and modifiable vascular risk factors: cigarette smoking, hypertension, diabetes, elevated total cholesterol, and excess body weight.

**Methods:**

Population attributable risks for CHD and stroke attributable to these risk factors individually were calculated using summary statistics obtained for prevalence of each risk factor specific to sex and to two age categories (<55 and ≥55 years) from a national survey in Indonesia. Age- and sex-specific relative risks for CHD and stroke associated with each of the five risk factors were derived from prospective data from the Asia-Pacific region.

**Results:**

Hypertension was the leading vascular risk factor, explaining 20%–25% of all CHD and 36%–42% of all strokes in both sexes and approximately one-third of all CHD and half of all strokes across younger and older age groups alike. Smoking in men explained a substantial proportion of vascular events (25% of CHD and 17% of strokes). However, given that these risk factors are likely to be strongly correlated, these population attributable risk proportions are likely to be overestimates and require verification from future studies that are able to take into account correlation between risk factors.

**Conclusions:**

Implementation of effective population-based prevention strategies aimed at reducing levels of major cardiovascular risk factors, especially blood pressure, total cholesterol, and smoking prevalence among men, could reduce the growing burden of CVD in the Indonesian population.

## INTRODUCTION

Indonesia is the fourth most populous country in the world, with a population of 250 million people, and has experienced rapid economic growth over the past couple of decades.^1^ With improved health financing, increased social mobilization, community empowerment, and prioritization of primary health care, the country has made visible achievements in several health outcomes. For example, life expectancies have steadily increased from 62 to 69 and 65 to 73 years for men and women, respectively, over the past decades, and rates of infant and maternal mortality have significantly decreased, in line with the Millennium Developmental Goals.^1^

As a likely consequence of improvements in the country’s economic development, Indonesia is experiencing a rapid epidemiological transition in terms of both its current and projected disease burden. While the existing burden of communicable diseases—and the likelihood of emerging diseases with epidemic or pandemic potential—is a key concern in Indonesia, the burden of disease related to non-communicable diseases has become a major public health issue.^2^^–^^5^ Roughly a third of all deaths in Indonesia are attributable to cardiovascular disease (CVD), with stroke and coronary heart disease (CHD) being the leading causes of mortality in the country.^3^^,^^5^^–^^8^

Similar to the situation in most other lower- and middle-income countries in the Asia-Pacific region, the prevalence of major cardiovascular risk factors, including excess body weight, diabetes, and elevated blood pressure, has risen in the Indonesian population.^9^^–^^17^ However, previous prevalence estimates were largely derived from demographic surveillance sites that were not nationally representative^9^^–^^11^ or were “guestimates” from modelling studies.^14^^–^^17^ The importance of obtaining reliable estimates of the prevalence of major and modifiable vascular risk factors cannot be overstated, as such data are the cornerstone of effective primary CVD prevention programs. Thus, we aimed to obtain representative prevalence estimates of cardiovascular risk factors as well as burden of CVD attributable to these risk factors using more reliable data sources than were used in previous studies.

## METHODS

### Risk factor assessment

The prevalence estimates included in this analysis were derived from a published report of Riskesdas 2013, a nationally representative survey conducted by the Republic of Indonesia’s Ministry of Health in 2013.^18^ The details about survey methodology, ethical clearance, data collection, validation, and analysis are explained in detail in the Riskesdas report 2013.^18^ Briefly, the survey included 1 027 763 subjects (approximately 300 000 households) at all ages from 33 districts of the country sampled through a probabilistic multi-stage process (eFigure 1). The Riskesdas households were selected based on the Population Census listing of 2010. The household selection process was determined by the Indonesian Central Bureau of Statistics, which provided a list of selected census buildings from selected census blocks using the technique of probability proportional to size (PPS). From each selected census block, 25 households were randomly selected, and from each selected household, all household members were considered as individual samples. The ethical clearance for the survey implementation was given by the Indonesian Ministry of Health’s National Institute of Health Research and Development Health Research Ethics Committee (IEC) (http://www.litbang.depkes.go.id/komisi-etik). For our analysis, we included information from subjects aged ≥15 years (*n* = 722 330). For collection of biomedical samples, a sub-sample of 1000 census blocks was randomly selected, and all household members of 25 selected households (approx. family size: 3.8) from each census block were included. Random blood samples were obtained from 40 250 participants aged ≥15 years who provided informed consent. Of these, values for fasting blood glucose and serum total cholesterol were available for 38 136 (94.7%) and 35 609 (88.4%) individuals, respectively. The age and sex distributions of participants in Riskesdas 2013 are presented in supplementary eTable 1. Of the 300 000 households targeted for the survey, 98.3% were successfully visited, giving an individual participant response rate of 93.0% (eTable 2).

Exposure status for each of the five risk factors was ascertained from either participants’ self-reports given in the standardized interview or measurements performed using standardized and quality-controlled procedures by trained personnel at the baseline examination.^18^ Smoking status (defined as occasionally/regularly or not) was based on self-report and was obtained from all participants aged ≥15 years. Blood pressure measurements (*n* = 661 367) were obtained only in those aged ≥18 years using a digital sphygmomanometer (Omron IA2; Omron, Kyoto, Japan). Hypertension was defined as systolic blood pressure ≥140 mm Hg or a diastolic blood pressure ≥90 mm Hg (Joint National Committee on Prevention, Detection, Evaluation, and Treatment of High Blood Pressure VII criteria).^19^ Diabetes was ascertained based on a self-report of a previous diagnosis of diabetes or use of anti-diabetic medication; presence of classic symptoms of hyperglycemia (polyuria, polydipsia, polyphagia, or unexplained weight loss) and a random plasma glucose ≥200 mg/dL (11.1 mmol/L); by fasting plasma glucose ≥126 mg/dL (7 mmol/L); or by 2-h serum glucose concentration ≥200 mg/dL after an oral glucose challenge.^20^^,^^21^ Elevated total cholesterol was conservatively defined as total cholesterol ≥5.2 mmol/L (200 mg/dL) rather than the more standard cut-point of 6.2 mmol/L, to include borderline high cholesterol individuals as well.^22^ Height was measured using an aluminium stadiometer capable of measuring to 0.1 cm, and weight was measured using a digital weighing machine measuring to the nearest 0.1 kg (Fesco, Jakarta, Indonesia). Excess body weight was defined in adults ≥18 years (*n* = 649 625) as a body mass index (BMI) value ≥25 kg/m^2^. The reported categorical values for the sex-specific prevalence for each of the cardiovascular risk factors were extracted from the survey report.^18^ The age-adjusted prevalence was calculated separately for younger (<55 years) and older (≥55 years) individuals. This age stratification was restricted according to the availability of risk estimates for these age groups.

### Relative risks of CHD and stroke

We obtained estimates of the relative risks (RRs) and their 95% confidence intervals (CIs) for CHD (fatal and non-fatal) and stroke (fatal and non-fatal) and by major stroke subtype (ischaemic and haemorrhagic) attributable to each of the five cardiovascular risk factors using Asian data from the Asia Pacific Cohort Studies Collaboration (APCSC).^23^ RRs for fatal events were also calculated. The definitions used for the risk factor exposures (eg, hypercholesterolemia ≥5.2 mmol/L) corresponded to the definitions used in Riskesdas 2013. The age- and sex-specific RRs were calculated using Cox proportional hazard models, which were adjusted for age and sex and stratified by study.

### Statistical analysis

The Levin formula (equation 1) for the population attributable risk proportion (PAR) for dichotomous exposures was used to obtain an estimate of the proportion of cases of CHD and stroke in the Indonesian population that can be attributed to exposure to a given cardiovascular risk factor.^24^

$$\text{PAR}=[{\text{P}}_{e}(\text{RR}-1)]/[{\text{P}}_{\text{e}}(\text{RR}-1)+1]$$ (1)

where P*_e_* is prevalence of an individual risk factor in the Indonesian general population and RR is age- and sex-adjusted and study-stratified RR.

We used the substitution method to calculate 95% CIs for PARs, which assumes that variability in the prevalence estimation is negligible compared to variability in the RR estimates.^25^ The standard errors of the RRs were extracted from the log-transformed 95% CIs available for each RR. Monte Carlo simulation^26^ of 10 000 iterations for each risk factor and for each sex and age category, from which we determined the 2.5 and 97.5 percentiles as empirical confidence limits for PAR, was performed using the Ersatz bootstrap add-in for Microsoft Excel (EpiGear, Sunrise Beach, Australia).^27^

## RESULTS

**Figure 1.**
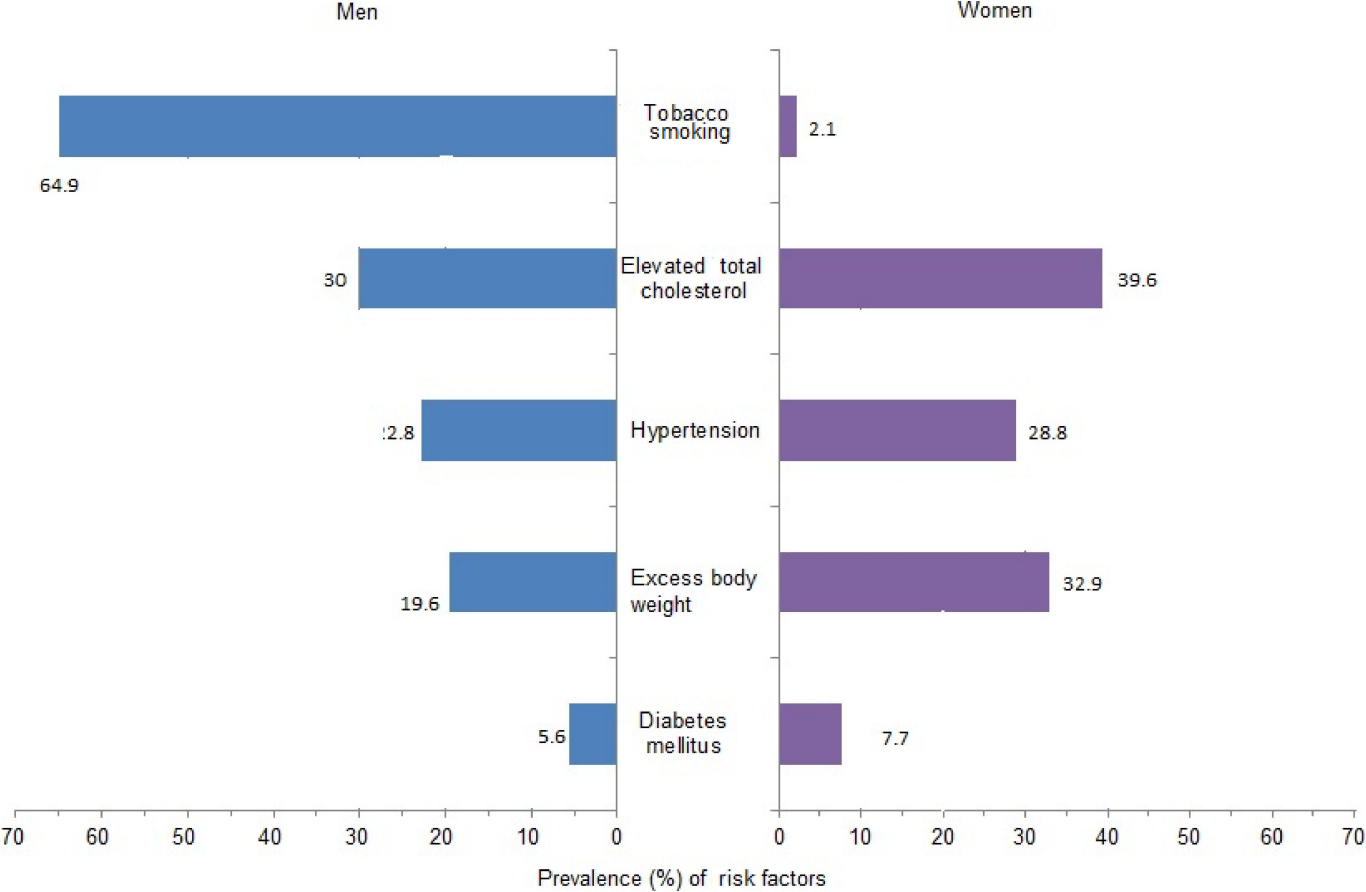
Prevalence (percentages) of selected cardiovascular risk factors in the Indonesian population. Prevalence of hypertension and overweight (BMI ≥25 kg/m^2^) was measured in individuals ≥18 years of age. Diabetes, smoking, and elevated total cholesterol (total serum cholesterol ≥5.2 mmol/L) were estimated for individuals ≥15 years old.

Of the risk factors included in this analysis, cigarette smoking was the most prevalent risk factor in men (64.9%), followed by elevated total cholesterol, hypertension, excess bodyweight, and diabetes (Figure 1). In women, the prevalence of smoking was low (2.1%), and elevated total cholesterol was the most prevalent risk factor (39.6%), followed by excess body weight, hypertension, and diabetes. The frequencies of all five risk factors differed significantly between men and women (all *P* < 0.001). Smoking and overweight were more common in younger (<55 years) than older (≥55 years) individuals (eFigure 2). By comparison, hypertension, diabetes, and high total cholesterol were more prevalent in older than younger individuals.

The PARs of CHD and stroke due to five major vascular risk factors in men and women are shown in Table 1. The percentage of CHD in men attributable to the risk factors ranged from 4.2% to 25.1%, with the greatest attributable fraction due to smoking and then hypertension (19.7%). In women, PARs ranged from 1.4% to 25.3%, with the highest attributable fraction due to hypertension and the lowest to smoking. Elevated total cholesterol also accounted for a large CHD burden in both sexes, accounting for 19% (95% CI, 14.2%–23.9%) of CHD events in men and 17.8% (95% CI, 7.2%–27.3%) in women. Similar results were also found for analyses involving only fatal CHD (eTable 3).

**Table 1.** Age- and sex-specific PAR percentages (95% CIs) associated with selected cardiovascular risk factors for incident CHD and stroke and its major subtypes in Indonesia

	Sex		Age group	
	
	Men	Women	<55 years	≥55 years
**All CHD**				
Smoking	25.1 (16.3, 32.8)	1.4 (0.7, 2.3)	20.3 (13.2, 27.9)	10.6 (5.0, 15.7)
Hypertension	19.7 (16.3, 23.6)	25.3 (18.1, 32.6)	32.9 (28.7, 37.5)	33.1 (26.6, 39.0)
Elevated total cholesterol	19.0 (14.2, 23.9)	17.8 (7.2, 27.3)	25.3 (20.0, 31.6)	17.7 (8.5, 25.7)
Excess body weight	9.4 (6.0, 12.7)	10.4 (2.2, 19.2)	19.6 (14.1, 25.1)	3.3 (−0.3, 7.9)
Diabetes	4.2 (2.3, 6.3)	10.7 (5.1, 17.6)	7.9 (5.3, 11.0)	8.4 (3.9, 13.8)

**All stroke**				
Smoking	16.7 (11.7, 21.8)	0.6 (0.2, 1.0)	11.1 (6.2, 15.4)	3.7 (0.5, 6.7)
Hypertension	37.2 (34.8, 39.6)	38.9 (34.7, 42.9)	49.0 (46.5, 51.6)	52.9 (49.6, 56.3)
Elevated total cholesterol	3.9 (1.1, 7.0)	6.9 (1.3, 12.5)	10.3 (7.0, 13.7)	2.2 (−3.7, 7.5)
Excess body weight	5.4 (3.2, 7.4)	8.2 (3.4, 12.5)	14.6 (11.1, 17.9)	0.4 (−1.8, 2.8)
Diabetes	3.8 (2.6, 5.0)	7.1 (3.9, 10.5)	6.3 (4.8, 7.9)	5.8 (2.8, 8.8)

**Ischaemic stroke**				
Smoking	25.1 (16.6, 33.3)	0.6 (0.1, 1.3)	14.4 (6.8, 23.1)	7.1 (2.1, 12.6)
Hypertension	29.3 (25.1, 33.0)	37.3 (30.8, 43.9)	38.2 (33.5, 43.4)	53.2 (47.6, 58.3)
Elevated total cholesterol	10.1 (5.6, 15.0)	10.1 (0.4, 19.9)	19.3 (13.6, 24.9)	2.6 (−5.4, 11.6)
Excess body weight	10.6 (7.2, 14.2)	15.1 (7.7, 22.2)	22.7 (17.4, 28.6)	4.4 (0.9, 8.1)
Diabetes	5.3 (3.6, 7.6)	6.0 (1.2, 12.7)	8.3 (5.7, 11.3)	7.3 (2.6, 12.7)

**Haemorrhagic stroke**				
Smoking	10.6 (1.2, 19.6)	0.6 (0.1, 1.4)	4.4 (−2.9, 10.9)	3.3 (−1.7, 8.8)
Hypertension	47.3 (43.1, 51.3)	46.6 (39.7, 53.2)	61.3 (57.1, 65.2)	56.2 (50.8, 61.6)
Elevated total cholesterol	−2.6 (−7.5, 2.3)	5.7 (−5.0, 16.2)	1.1 (−4.2, 6.6)	2.8 (−7.5, 12.9)
Excess body weight	3.8 (0.7, 7.6)	1.9 (−5.4, 9.6)	10.9 (5.1, 16.8)	−1.2 (−5.0, 3.1)
Diabetes	2.3 (0.5, 4.3)	8.4 (2.0, 17.1)	4.9 (2.5, 7.8)	2.7 (−2.6, 9.7)

CHD, coronary heart disease; CI, confidence interval; PAR, population attributable risk.

When comparing older versus younger individuals (men and women combined), hypertension conferred the highest PAR in both age groups (Table 1) and accounted for approximately 33% of all CHD in both age groups.

For stroke, hypertension was the leading risk factor in both sexes, accounting for 37.2% of stroke episodes in men and 38.9% of strokes in women (Table 1). When distinguishing between stroke subtypes, notable differences in the attributable fraction were observed: roughly half of all haemorrhagic strokes in women and men, and in younger and older individuals, were attributable to hypertension (Table 1). For ischemic stroke, hypertension explained about one-third of all events in women and men and in those aged <55 years, while it accounted for more than half of all ischemic strokes in those aged ≥55 years. Smoking explained 16% of all strokes in men and 0.6% of strokes in women. Elevated total cholesterol accounted for approximately 10% of all ischemic strokes in both sexes (and was unrelated to haemorrhagic stroke). The attributable burden of ischemic stroke due to elevated total cholesterol, excess body weight, and smoking was higher in younger versus older individuals (eTable 2). The results were highly comparable for fatal strokes (eTable 3).

## DISCUSSION

Contemporary estimates indicate that CHD and stroke are already the leading causes of death in the Indonesian population, accounting for more than 30% (0.47 million) of all mortality.^8^ Findings from the current study suggest that a substantial amount of the burden of CHD and stroke in the Indonesian population is attributable to major and modifiable (and therefore, preventable) vascular risk factors, most notably elevated blood pressure, total cholesterol, and cigarette smoking (but only in men). However, we observed some notable disparities in the vascular burden due to specific risk factors, as well as important sex and age differences. For example, in men, hypertension was the leading risk factor, attributing to 35%–40% of all stroke and 20% of all CHD, followed by smoking, which accounts for a quarter of all CHD and 16% of all stroke. However, in women, hypertension alone was the dominant risk factor, accounting for 25% of all CHD and 39% of all stroke events, while each of the remaining risk factors explained between 2% and 20% of the vascular burden. Across both younger and older age groups, hypertension was the single leading risk factor, accounting for 33% of all CHD and 50% of all stroke episodes. In comparison, smoking, high cholesterol, and excess body weight were responsible for a higher vascular burden in younger compared with older individuals.

A key strength of the current study was that it used nationally representative data from the Riskesdas 2013 survey to obtain age- and sex-specific estimates of the prevalence of these major and modifiable vascular risk factors in the Indonesian adult population. In men, smoking was the most prevalent risk factor; approximately two-thirds of men were current smokers compared with only 2% of women, which wholly explains the dominance of smoking in the PAF estimates observed in men. This sex-disparity in smoking rates is a common pattern in most lower- and middle-income countries in the Asia-Pacific region, especially in neighbouring countries like Malaysia and the Philippines.^28^^,^^29^ By comparison, elevated cholesterol and excess body weight were more prevalent in women compared to men; this greater prevalence of excess body weight explains the greater proportion of vascular events in women than in men. Although the prevalence of diabetes was similar between the sexes (5.6% in men and 7.7% in women), diabetes explained nearly twice the number of all CHD and strokes in women than in men due to the relationship between diabetes with vascular outcomes being significantly stronger in women than in men.^30^^,^^31^

In general, our results are in agreement with previous findings.^3^^,^^32^^–^^36^ For example, high blood pressure, tobacco smoking, high BMI, and high total cholesterol were among the major risk factors for vascular disease in Indonesia identified by the Global Burden of Disease 2010 report,^3^ the estimates from which were largely based on data from the 2001 STEPS survey and additional small, non-representative surveys.^9^^,^^14^^,^^16^^,^^37^^–^^39^ In comparison, in the current study, we used the age- and sex-specific prevalence estimates for risk factors extracted from the latest round of the basic health research Riskesdas 2013 survey. However, the lack of prospective cohort studies specific to the Indonesian population meant that relative risk estimates were sourced from the APCSC. Nonetheless, this is likely to have afforded more reliable estimates of the relationships of each of the risk factors with vascular outcomes in the Indonesian population than if we had used relative risk estimates derived from non-Asian studies.^23^^,^^40^ The dominance of hypertension as the preeminent vascular risk factor in the Indonesian adult population is consistent with previous reports from large-scale initiatives, including the APCSC^41^ and INTERHEART.^33^ Ezzati et al previously reported that 45% of global CHD is attributable to hypertension, which is double the proportion that we observed in the current study.^36^ This discrepancy is likely due to the use of 115 mm Hg as the lower threshold for blood pressure used for risk estimation in that study compared to the much more conservative definition of hypertension used in Riskesdas 2013 (140/90 mm Hg). Moreover, in the current study, individuals taking antihypertensive medication were not included in the definition of hypertension, nor were individuals with ‘pre-hypertension’ (ie, systolic blood pressure between 120–139 mm Hg), which would have necessarily resulted in an underestimation of the true burden of elevated blood pressure on vascular disease.

The study has several limitations. First, the five risk factors included in the model were limited to those that were reported in Riskesdas 2013 and for which we could obtain estimates of effect from the APCSC database; consequently, we did not examine the contribution of other established risk factors, such as diet and physical activity, to the burden of vascular disease. Second, the prevalence of risk factors was reported according to pre-defined categories, and we did not have access to the individual-level data that would have facilitated the derivation of standard errors around the prevalence estimates, which would have allowed the calculation of the PARs for CHD and stroke attributable to all five risk factors combined or within specific sex-age groups. However, we estimated the posterior probability of PAR estimates for individual risk factors through Monte Carlo simulation (10 000 iterations) using the substitution method^25^ (assuming that the variability in prevalence estimates is minimal compared to that of the relative risk). Related to this limitation, we were unable to take into account possible correlation between the five risk factors, as we did not have access to individual-level data. Consequently, contained within the PAR estimates was the implicit assumption that the five risk factors acted independently to increase vascular risk, which is highly unlikely. For example, BMI is known to have both direct and indirect effects on vascular risk (the latter being partly driven by the impact of BMI on risk of diabetes). Hence, the PAR estimates that we present do not take into consideration either the clustering or the non-independence of risk factors, so they are likely to be overestimates. Alternate case-based estimation methods would be more appropriate in this situation, as such methods utilize the adjusted attributable fraction derived from the relative risk and prevalence of exposure among the diseased and produce internally valid estimates when confounding exists.^42^^,^^43^ Additional alternatives, such as the stratification-based adjustment method, sequential attributable fraction, and regression model-based approaches, would have been useful for dealing with multiple risk factors that have direct and indirect effects on the exposure-effect association.^44^^,^^45^ However, as we did not have individual-level data, we were unable to apply any of these alternate, and more appropriate, methods for estimation of the PAFs. Future studies should be carried out using individual-level data, which will enable the modelling of relationships between risk factors and the outcome and allow calculation of flexible and efficient estimation of the adjusted PAF, taking into account several categorical or continuous risk factors or confounding factors with or without their interactions and allowing for the analysis of potential effect modification.^45^

Recently, the Indonesian government has operationalized several initiatives to combat the growing cardiovascular epidemic, including training primary care workers and family practitioners to assess and manage cardiovascular risk, enacting legislation for pictorial warnings to cover a significant area (40%) on tobacco product packages,^46^ developing of non-smoking areas, and empowering communities to control risk factors for cardiovascular disease through the establishment of ‘Integrated Health Training Posts (*Posbindu*) in NCD’. Whether these and other measures^2^ will be effective in reducing Indonesia’s cardiovascular burden will depend, in part, on the coverage of the interventions across the Indonesian archipelago, whether the policies are enforced, and the cultural acceptability of the interventions across all population sub-groups.

In summary, although our findings are likely to have overestimated the vascular disease burden due to five major and modifiable risk factors, they support the viewpoint that a substantial proportion of premature cardiovascular morbidity and mortality in the Indonesian population is preventable. Indonesia already has a high unmet need for cardiovascular care^47^; the present analysis provides useful and timely information that could be used for the identification and prioritization of public health interventions for the primary prevention of vascular disease in the overall Indonesian population and within specific subgroups. Ultimately, primary prevention strategies that focus on lowering mean levels of blood pressure at the population level and reducing the prevalence of smoking in men are likely to have a profound impact on reducing the burden of vascular disease in the Indonesian population.

## ONLINE ONLY MATERIALS

eTable 1. Distribution of Riskesdas sample (age ≥15 years) by age and sex compared with the national census population.

eTable 2. Distribution of census blocks, households, and individual household member and their response rates in different provinces of Indonesia, 2013.

eTable 3. Sex- and age-specific PAR (%) associated with selected cardiovascular risk factors for fatal CHD and stroke in the Indonesian population.

eFigure 1. Sampling strategy used for selection of households and individual participants in the Riskesdas survey.

eFigure 2. Age-standardized prevalence (%) of selected cardiovascular risk factors in the Riskesdas survey.
