# Supplementary material for: The Burden of Cardiovascular Disease Attributable to Major Modifiable Risk Factors in Indonesia
Source: J Epidemiol. 2016 Oct 5;26(10):515–21. doi: 10.2188/jea.JE20150178 (PMC5037248; doi:10.2188/jea.JE20150178)
Supplement: eTable 1. [file je-26-515-s001.pdf]

eTable 1. Distribution of Riskesdas sample (age ≥15 years) by age and sex compared with the national census population

Age groups, years	Male			Female				
	Number (a)	Percentage (a/[a+b])	National sample proportion (%)	Number (b)	Percentage (b/[a+b])	National sample proportion (%)	Total Sample (overall %) (a+b)	Overall % in the census population
15-19	44,274	51.35	50.83	41,948	48.65	49.17	86,222 (11.93)	12.35
20-24	31,796	50.57	49.71	31,082	49.43	50.29	62,878 (8.70)	11.77
25-29	29,497	45.45	49.89	35,402	54.55	50.11	64,899 (8.98)	12.61
30-34	35,303	45.25	50.17	42,723	54.75	49.83	78,026 (10.80)	11.73
35-39	36,807	46.16	50.46	42,924	53.84	49.54	79,731 (11.03)	10.95
40-44	38,811	47.96	50.36	42,107	52.04	49.64	80,918 (11.20)	9.78
45-49	33,626	48.04	50.09	36,370	51.96	49.91	69,996 (9.69)	8.31
50-54	30,452	49.92	50.74	30,549	50.08	49.26	61,001 (8.44)	6.84
55-59	23,504	49.51	52.08	23,965	50.49	47.92	47,469 (6.57)	5.00
60-64	17,406	50.59	48.31	17,000	49.41	51.69	34,406 (4.73)	3.58
65-69	10,400	47.80	47.40	11,358	52.20	52.60	21,758 (3.01)	2.78
70-74	8,419	47.86	44.31	9,173	52.14	55.69	17,592 (2.43)	2.04
75+	7,529	43.19	41.89	9,905	56.81	58.11	17,434 (2.41)	2.27
Total	347,824	48.15		374,506	51.85		722,330 (100)	100.00
