# Supplementary material for: The Burden of Cardiovascular Disease Attributable to Major Modifiable Risk Factors in Indonesia
Source: J Epidemiol. 2016 Oct 5;26(10):515–21. doi: 10.2188/jea.JE20150178 (PMC5037248; doi:10.2188/jea.JE20150178)
Supplement: eTable 2. [file je-26-515-s002.pdf]

eTable 2. Distribution of census blocks, households, and individual household member and their response rates in different provinces of Indonesia, 2013

Serial number[a]	Provinces	Census blocks		Households			Individual household members		
		Number visited	Response rate (%)	Total number	Number visited	Response rate (%)	Total Number	Information recorded	Response rate (%)
1	Aceh	467	100	11,675	11,617	99.5	44,368	40,951	92.3
2	North Sumatra	756	100	18,900	18,693	98.9	75,547	72,935	96.5
3	West Sumatra	407	100	10,175	10,023	98.5	38,913	36,955	95.0
4	Riau	305	100	7,625	7,520	98.6	29,621	28,017	94.6
5	Jambi	250	100	6,250	6,189	99.0	23,056	22,605	98.0
6	South Sumatra	383	100	9,575	9,549	99.7	38,089	37,393	98.2
7	Bengkulu	204	100	5,100	5,072	99.5	18,897	18,154	96.1
8	Lampung	373	100	9,325	9,268	99.4	33,440	32,745	97.9
9	Bangka Belitung	144	100	3,600	3,569	99.1	12,759	11,765	92.2
10	Riau Islands	145	100	3,625	3,546	97.8	12,837	11,844	92.3
11	Jakarta	208	99.5	5,225	4,684	89.6	16,343	13,766	84.2
12	West Java	958	100	23,950	23,694	98.9	83,522	77,701	93.0
13	Central Java	1,098	100	27,450	27,255	99.3	93,650	85,310	91.1
14	DI Yogyakarta	150	100	3,750	3,704	98.8	12,100	11,104	91.8
15	East Java	1,197	100	29,925	29,717	99.3	104,483	97,339	93.2
16	Banten	271	100	6,775	6,679	98.6	26,277	24,247	92.3
17	Bali	231	100	5,775	5,761	99.8	21,508	20,403	94.9
18	West Nusa Tenggara	254	100	6,350	6,339	99.8	23,486	22,256	94.8
19	East Nusa Tenggara	436	100	10,900	10,747	98.6	46,206	43,732	94.6
20	West Kalimantan	324	100	8,100	8,000	98.8	30,670	29,050	94.7
21	Central Kalimantan	277	100	6,925	6,773	97.8	24,021	22,284	92.8
22	South Kalimantan	300	100	7,500	7,298	97.3	26,248	24,532	93.5
23	East Kalimantan	293	100	7,325	6,950	94.9	25,747	23,931	92.9
24	North Sulawesi	298	100	7,450	7,395	99.3	25,293	24,047	95.1
25	Central Sulawesi	240	100	6,000	5,800	96.7	23,185	21,128	91.1
26	South Sulawesi	553	100	13,825	13,598	98.4	52,425	48,129	91.8
27	Southeast Sulawesi	237	100	5,925	5,908	99.7	24,387	22,766	93.4
28	Gorontalo	122	100	3,050	3,029	99.3	12,029	11,242	93.5
29	West Sulawesi	106	100	2,650	2,628	99.2	10,817	9,952	92.0
30	Moluccas	199	100	4,975	4,945	99.4	22,301	19,665	88.2
31	North Maluku	161	100	4,025	3,913	97.2	17,301	15,755	91.1
32	West Papua	160	99.4	4,025	3,836	95.3	15,288	13,046	85.3
33	Papuan	479	97.6	12,275	11,260	91.7	40,779	33,014	81.0
	Indonesia	11,986	99.9	300,000	294,959	98.3	1,105,593	1,027,763	93.0

[a] Extracted from the report of national basic health research (Riskesdas 2013)[18]
