# Supplementary material for: The Burden of Cardiovascular Disease Attributable to Major Modifiable Risk Factors in Indonesia
Source: J Epidemiol. 2016 Oct 5;26(10):515–21. doi: 10.2188/jea.JE20150178 (PMC5037248; doi:10.2188/jea.JE20150178)
Supplement: eTable 3. [file je-26-515-s003.pdf]

eTable 3. Sex- and age-specific PAR (%) associated with selected cardiovascular risk factors for fatal CHD and stroke in the Indonesian population

	Smoking	Hypertension	Elevated total cholesterol	Excess body weight	Diabetes
**Men**					
Fatal CHD	28.0 (20.1, 35.4)	20.1 (16.0, 23.8)	13.0 (7.4, 18.5)	7.7 (4.0, 11.8)	6.4 (3.7, 10.1)
Fatal Stroke	17.2 (12.0, 22.5)	40.1 (37.4, 42.8)	3.5 (-0.2, 7.1)	2.5 (0.1, 4.9)	4.2 (2.4, 6.4)
**Women**					
Fatal CHD	1.3 (0.8, 1.7)	24.1 (19.8, 28.5)	16.7 (9.2, 23.2)	12.1 (6.4, 18.3)	12.0 (5.5, 20.3)
Fatal Stroke	0.7 (0.4, 0.9)	45.8 (42.6, 48.6)	4.4 (0.3, 8.9)	4.0 (0.3, 8.0)	7.2 (3.2, 12.2)
**<55 years**					
Fatal CHD	22.7 (12.8, 33.5)	36.2 (29.9, 42.4)	17.9 (9.7, 27.1)	17.4 (8.7, 27.0)	11.0 (6.5, 16.7)
Fatal Stroke	13.1 (6.2, 19.6)	59.1 (55.4, 62.7)	7.2 (1.4, 13.4)	10.2 (4.1, 16.7)	8.3 (5.0, 12.1)
**≥55 years**					
Fatal CHD	8.4 (2.5, 14.5)	33.1 (25.4, 40.1)	15.7 (4.5, 15.6)	3.7 (-0.7, 8.6)	12.5 (6.7, 19.4)
Fatal Stroke	2.9 (-0.5, 6.4)	55.4 (51.7, 58.9)	2.7 (-4.3, 9.3)	-1.4 (-4.1, 1.5)	6.0 (2.2, 10.3)

CHD, coronary heart disease; PAR, population attributable risk.
