# Supplementary material for: The Burden of Cardiovascular Disease Attributable to Major Modifiable Risk Factors in Indonesia
Source: J Epidemiol. 2016 Oct 5;26(10):515–21. doi: 10.2188/jea.JE20150178 (PMC5037248; doi:10.2188/jea.JE20150178)
Supplement: eFigure 1. [file je-26-515-s004.pdf]

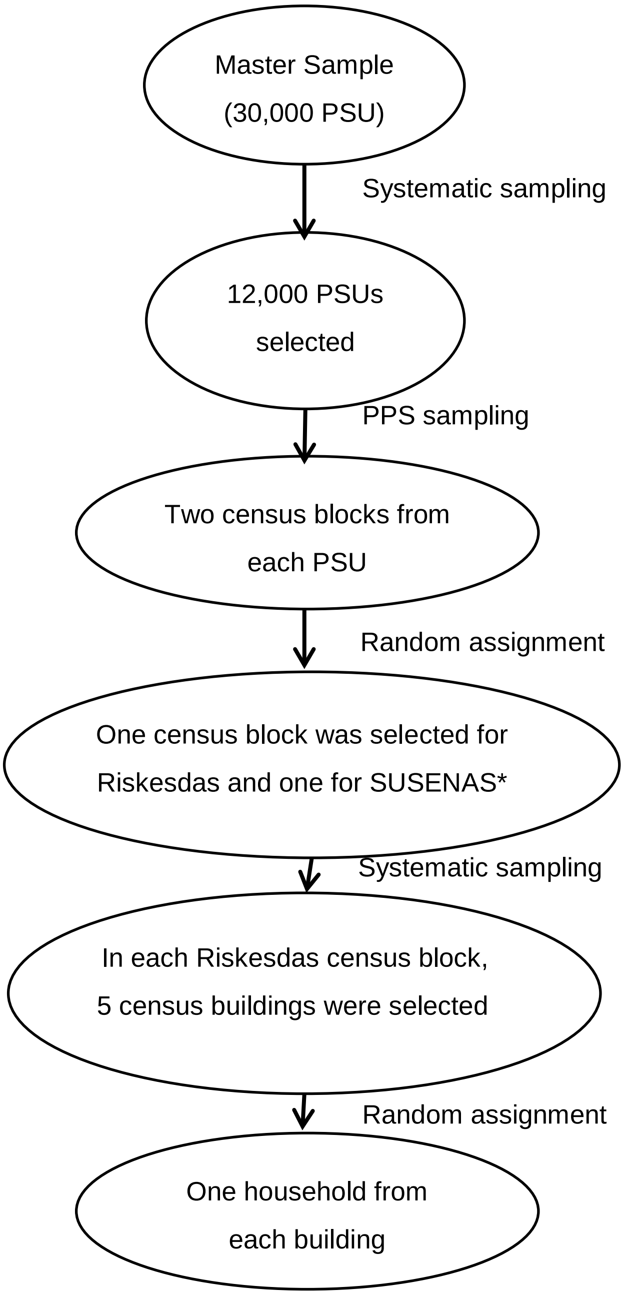
Master Sample
(30,000 PSU)
Systematic sampling
12,000 PSUs
selected
PPS sampling
Two census blocks from
each PSU
Random assignment
One census block was selected for
Riskesdas and one for SUSENAS*
Systematic sampling
In each Riskesdas census block,
5 census buildings were selected
Random assignment
One household from
each building

eFigure 1. Sampling strategy used for selection of households and individual participants in the Riskesdas survey. Primary sampling units (PSUs) were selected using the probability proportional to size method across all districts based on the number of households in the 2010 census population. Each PSU was comprised of a different number of census blocks depending upon population size and urban and rural difference. For a targeted selection of 300,000 households, 12,000 PSUs were selected from the master sampling frame proportionate to urban and rural area distribution. For collection of biomedical samples, a sub-sample of 1,000 census blocks was randomly selected, and all members of 25 selected households in each block (approximate family size: 3.8) were included for biomedical sample collection.

*SUSENAS- Indonesia’s National Socio-Economic Survey
