# Supplementary material for: The Burden of Cardiovascular Disease Attributable to Major Modifiable Risk Factors in Indonesia
Source: J Epidemiol. 2016 Oct 5;26(10):515–21. doi: 10.2188/jea.JE20150178 (PMC5037248; doi:10.2188/jea.JE20150178)
Supplement: eFigure 2. [file je-26-515-s005.pdf]

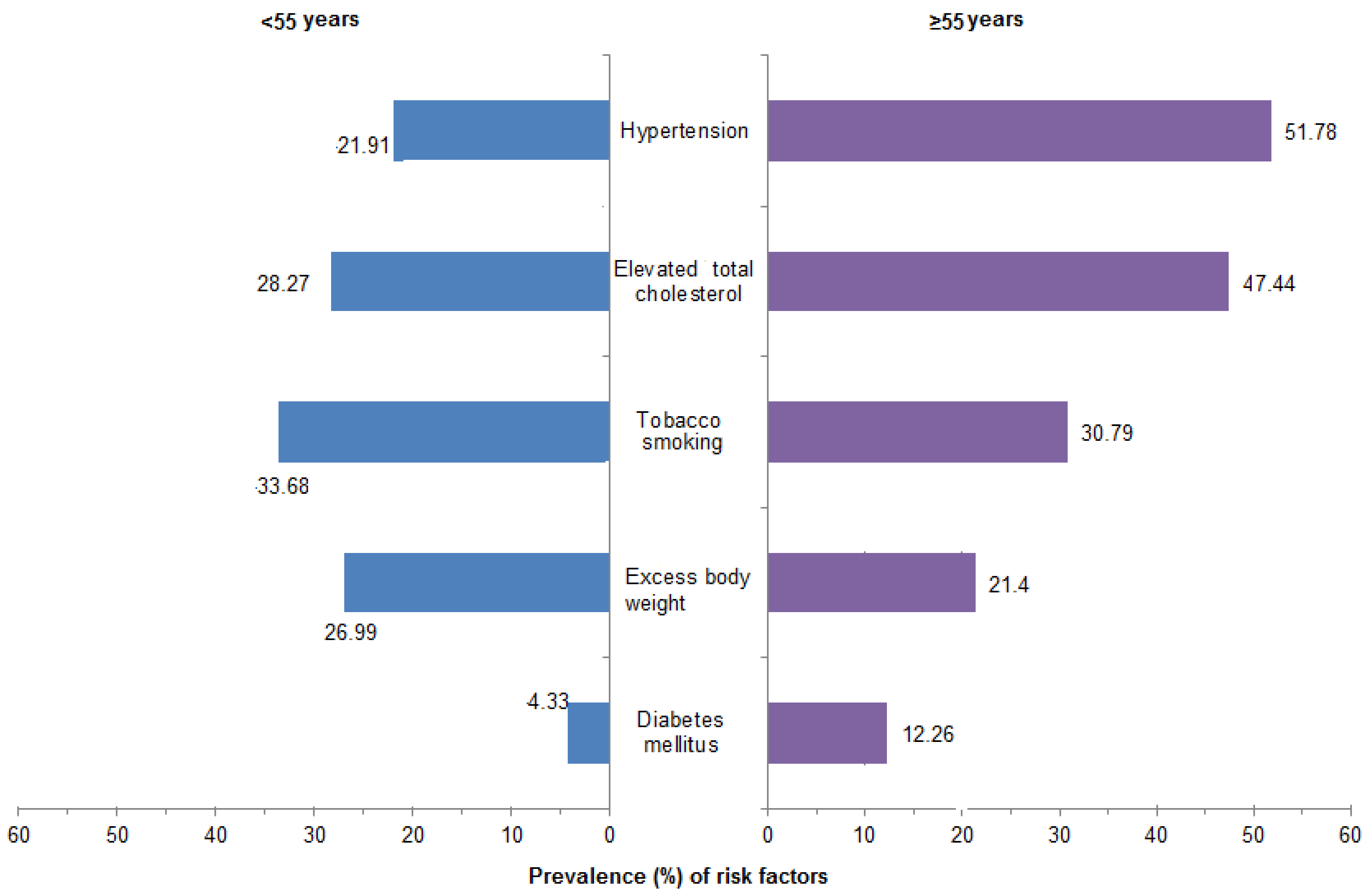

eFigure 2. Age-standardized prevalence (%) of selected cardiovascular risk factors in the Riskesdas survey
